# Results from a randomized trial combining trastuzumab with a peptide vaccine suggest a role for HER2-targeted therapy in triple-negative breast cancer

**DOI:** 10.18632/oncotarget.27998

**Published:** 2021-11-09

**Authors:** Anne E. O’Shea, Guy T. Clifton, George E. Peoples

**Keywords:** HER2, peptide vaccines, nelipepimut-S, triple-negative breast cancer

HER2 has been identified as a target antigen for cancer vaccines and immunotherapy due to its over-expression and oncogenic potentiation in a subset of breast and gastroesophageal cancers. However, even though HER2 contains immunogenic epitopes, to date, neither HER2-targeted vaccines alone nor passive, monoclonal antibody-based HER-directed therapy have shown clinical benefit in patients with HER2 low-expressing tumors (IHC 1-2+) in large, randomized controlled trials [1, 2] Preclinical data allude to a synergistic effect of HER2-targeted therapy, with or without concomitant cytotoxic chemotherapy, when combined with either checkpoint inhibitors, or cancer vaccine immunotherapy [3–6]. These encouraging preclinical findings together with an established and favorable safety profile provided the impetus for the multicenter, prospective, randomized, single-blinded, placebo-controlled phase IIb trial of trastuzumab and the HER2-derived peptide vaccine nelipepimut-S (NPS) with granulocyte-macrophage colony-stimulating factor (GM-CSF) versus trastuzumab and placebo with GM-CSF in HER2 low-expressing tumors [7]. This trial, along with ongoing clinical trials evaluating the combination of HER-directed therapies with immune checkpoint inhibitors, will help us better appreciate the value of combining immunotherapy with HER2-directed therapies and determine the subset of patients who stand to benefit most from these combinations.

The phase II trial randomized 275 patients with HLA-specific, HER2 low-expressing (IHC 1+ or 2+) breast cancer at high risk of recurrence (node positive or estrogen receptor negative/progesterone receptor negative) rendered disease-free by surgery and adjuvant/neoadjuvant therapies 1:1 to receive either trastuzumab and NPS + GM-CSF or trastuzumab and placebo + GM-CSF. Patients were treated with trastuzumab for one year while receiving peptide vaccine or control. The vaccine series consisted of six inoculations delivered every three weeks followed by four booster inoculations delivered every six months. The primary study outcome was 24-month disease-free survival (DFS). Secondary outcomes included 36-month DFS, safety analysis, and immunologic response. At a median follow-up of 25.7 months, no significant difference was noted in 24-month DFS between the vaccine and placebo treatment groups in the intention-to-treat population (89.8% vs. 83.8%, respectively, *p* = 0.18). However, among patients with triple negative breast cancer (TNBC; ER-negative, PR-negative, and HER2-negative), at a median follow-up of 26.1 months, patients who received NPS had significantly improved 24-month DFS when compared to placebo (92.6% vs. 70.2%, respectively, *p* = 0.01) [7].

While this clinical trial failed to demonstrate improvement in DFS in all patients with low-expressing HER2 breast cancer at high risk of recurrence, it was designed and initiated before the differences in immunogenicity between breast cancer subtypes were widely recognized. It is now well-established that breast cancer subtypes differ in their interaction with the immune system and their susceptibility to immunotherapy. Two subtypes in particular, TNBC and HER2-positive breast cancer, carry a greater mutational load, higher expression of PD-L1, and more immune infiltrate compared to hormone receptor positive breast cancers [8–11]. As such, these breast cancer subtypes may be more responsive to immunotherapeutic approaches. Therefore, the results of the planned subgroup analysis of TNBC, in which patients experienced a significant improvement in DFS with the addition of NPS to trastuzumab compared with trastuzumab alone, fit with our understanding of TNBC biology and warrant further investigation.

Trastuzumab, an IgG1 kappa monoclonal antibody against the HER2 receptor, has been found to effect both the innate and adaptive immune system. Trastuzumab facilitates antibody-dependent cell-mediated cytotoxicity (ADCC), a function of the innate immune system; however, this ability depends on a competent adaptive immune response [12, 13]. Furthermore, trastuzumab promotes Fc receptor-mediated uptake and cross-presentation of soluble HER2 antigens by dendritic cells resulting in immune priming of naïve CD8+ T cells into HER2-specific cytotoxic T lymphocytes, an effect which may be amplified by combining with a HER2-directed vaccine [3]. Finally, treatment with the HER-directed therapy, trastuzumab, has demonstrated upregulation of PD-L1 expression on breast cancer cells in transgenic mouse models, and it is hypothesized that this serves as a possible means of trastuzumab resistance [14]. These immunologic effects of trastuzumab provide the justification for combining immunomodulators such as PD-1/PD-L1 and/or CTLA-4 targeted therapy or HER2-derived peptide vaccines with the HER-directed antibody therapy in an effort to optimize anti-tumor responses.

Preclinical breast cancer models treated with trastuzumab, trastuzumab emtansine (T-DM1), or trastuzumab deruxtecan combined with PD-1 and/or CTLA-4 targeted therapy have shown improved therapeutic effectiveness [4–6]. At this time, several ongoing clinical trials are working to evaluate the safety and efficacy of these drug combinations in HER2 over-expressing breast cancer, trastuzumab resistant metastatic breast cancer, HER2 under-expressing breast and urothelial cancer, and small cell lung cancer [15–19]. So far, the combination has been demonstrated to be safe with at least improvements in progression free survival (PFS) in PD-L1 positive subsets of patients [20].

Some of the immunologic mechanisms of action described above may allow trastuzumab to work synergistically with the HER2-targeted peptide vaccine, NPS, to bring new treatment options to TNBC patients. There are also multiple promising ongoing trials that are evaluating other immunotherapies combined with trastuzumab. Hopefully, these and future studies will expand treatment options for the subsets of breast cancer patients who will benefit most from the combination of HER2-directed therapy and immunotherapy.
